# Comparison of ultrasound quality assurance phantom measurements from matched and mixed scanner‐transducer combinations

**DOI:** 10.1120/jacmp.v4i3.2521

**Published:** 2003-06-01

**Authors:** D. J. Tradup, N. J. Hangiandreou, J. P. Taubel

**Affiliations:** ^1^ Department of Radiology Mayo Clinic 200 First Street SW Rochester Minnesota 55905

**Keywords:** ultrasound imaging, quality assurance testing, image quality assessment

## Abstract

Our goal in this work was to compare the results of common phantom tests made using matched and mixed ultrasound (US) scanner‐transducer combinations. Sets of common US quality assurance (QA) measurements were made using matched US scanner‐transducer combinations (i.e., transducers purchased for use with a particular scanner), as well as unmatched (mixed) combinations. Measurements of vertical and horizontal distance accuracy, and depth of penetration were performed using three common transducer types. Means, standard deviations, and differences between the mean mix and match measurements divided by the standard deviation (match‐mix difference, or MMD), and two‐sided, paired *t*‐tests were computed for the groups of mixed and matched measurements. MMDs for vertical and horizontal distance accuracy test results were less than 0.87 in all cases, well below our threshold value of 2.0, which indicates that a significant difference exists. MMDs for the depth of penetration measurements were less than 1.50, again below the threshold value. These results suggest that all of the mixed and matched data sets were very similar. The more sensitive *t*‐tests indicate statistically significant differences in only 2 of the 18 pairs of data sets. In conclusion, this study suggests that QA measurements generated by mixed or matched scanner‐transducer combinations are very comparable. The ability to obtain QA phantom test data from mixed scanner‐transducer combinations reduces the time required for US QA testing.

PACS number(s): 87.57.–s, 87.62.+n

## INTRODUCTION

With continued increasing use of ultrasound and advances in technology comes the demand for growth and change of ultrasound (US) quality assurance (QA) programs. In our radiology US practice, we generate over 7000 ultrasound exams or ultrasound related procedures each month. Ultrasound imaging is done in five centers on the medical campus separated by up to a mile. In addition, there are two off‐campus locations where mobile ultrasound is performed. Our ultrasound equipment inventory consists of 44 ultrasound scanners and 251 transducers. Each scanner was purchased with anywhere from one to ten transducers. Most of the transducers remain matched with the ultrasound scanner for which they were initially purchased. However, we have found that many transducers have a tendency to migrate around the department. Certain imaging locations generate more exams of specific types than others, so more transducers of specific types tend to accumulate in these areas. As a result, a significant amount of clinical scanning in our practice occurs using unmatched (i.e., mixed) scanner‐transducer combinations.

Design and implementation of the US QA program in our department is directed by a medical physicist. The continuing development of our program considers input from many sources, including the American Association of Physicists in Medicine,^1^ other articles in the literature,^2,^, ^3^ the American College of Radiology accreditation requirements,^4^ previously implemented QA programs, as well as results from our own investigations (such as the current study). Our basic QA phantom tests include a visual evaluation of image uniformity and quantitative evaluations of vertical distance accuracy (VDA), horizontal distance accuracy (HDA), and maximum depth of penetration (DOP). The complete set of QA tests is performed for each multifrequency transducer using two different frequencies every six months.

A key motivator for the current study was the amount time we found was needed to test our large and growing ultrasound equipment inventory. Initially, our ultrasound quality assurance program strictly followed the common guideline of only testing each ultrasound transducer matched with the ultrasound scanner for which it was purchased.^1^ Due to the number of mixed scanner‐transducer combinations found in many scan rooms, a significant amount of time was required to locate and collect these transducers from across campus, transport them back to the matched scanner for QA testing, and then return them to the imaging area where they were needed. Not only does this process consume significant time on the part of the QA staff, but the time that the transducers are not available at their clinical imaging sites is an important consideration as well. We designed this study comparing matched and mixed QA test data to determine the practical importance of obtaining only matched data for QA purposes. An initial preliminary study was performed.^5^ The current study was improved by focussing only on tests in our QA program, including larger data sets, and employing more sensitive statistical testing.

## MATERIALS AND METHODS

We collected and analyzed data obtained from Acuson Sequoia US scanners (Siemens Medical Solutions, Mountain View, CA), using three common ultrasound transducer types: the 8L5 linear array, the 8C4w curvilinear array, and the 4V1 vector array. Ultrasound QA measurements were made using ten ultrasound scanners and ten transducers of each type, each operated at two different frequencies. Measurements were made using each transducer matched with the scanner with which it was purchased (“matched”), as well as with one other scanner (“mixed”). Ten matched and ten mixed ultrasound scanner‐transducer combinations were used. All QA tests were performed using a commercial US phantom (RMI Model 405GS LE, Gammex RMI, Middleton, WI).

We evaluated VDA, HDA, and DOP as described in our US QA program documentation, which follows the general test descriptions given in Ref. 1. The standard scanner presets prescribed in our QA procedures were used, along with plastic templates to provide reproducible TGC (time gain compensation) control positions. Prior to scanning, room lights were dimmed and our standard scanner display monitor contrast and brightness settings were verified. A single frozen image for each transducer frequency was sufficient for making VDA, HDA, and DOP measurements. The vertical distance accuracy test was performed by measuring the distance between two line targets in a vertical column in the phantom using the US scanner calipers, and then comparing this with the distance provided in the phantom specification. The horizontal distance accuracy test was performed in a similar fashion, but measuring the distance between two line targets in a horizontal row in the phantom. The phantom documentation does not provide tolerances on the line target separations; however, these are likely on the order of the line target diameter (0.1 mm). The speed of sound in the phantom is specified as 1540±10 m/s. The maximum depth of penetration measurement was performed by viewing the speckle pattern in an image of a uniform section of the phantom, and using the calipers to subjectively estimate the greatest depth from which “usable echo information” was obtained.^1^

Groups of data in both the matched and mixed data sets were further separated according to QA measurement type (VDA, HDA, or DOP), transducer (8L5, 8C4w, or 4V1), and transducer frequency. The mean and standard deviation of each data group was computed. For the VDA and HDA data groups, the absolute measurement error was also computed by taking the difference between the mean US measurement and the known actual distance between the line targets, and dividing by the actual distance. Measurement error values that are negative indicate measured distances which are smaller than the actual distances. For each pair of corresponding data groups (each pair consisting of one group of matched measurements and one group of mixed measurements), we computed the difference between the mean mix and match measurements divided by the standard deviation (match‐mix difference, or MMD), and also performed two‐sided, paired *t*‐tests. The MMD parameter is analogous to a measurement of signal‐to‐noise ratio (SNR), which can be defined as the difference in the mean values divided by the average of the standard deviations. We chose to define a separate parameter (MMD) in order to avoid possible confusion with other, more standard applications of the terms “signal‐to‐noise ratio” and “SNR.” MMD values which are negative indicate that the mean match measurement was smaller than the corresponding mean mix measurement. We compared the absolute MMD value to a threshold of 2 and judged that if MMD <2, the mix and match data sets are substantially the same. If MMD>2, we conclude that the mix and match data sets are different from one another. This is similar to the threshold SNR discussion presented in Chapter 1 of Ref. 6, except that we have chosen a relatively smaller threshold value in order to increase our sensitivity to possible differences between the mix and match data sets. Lower *t*‐test *P*‐values indicate greater likelihood that the mix and match data sets are actually different from one another. In this study, we used a 95% confidence level in our statistical testing, so if the *P*‐value is greater than 0.05 we judge the mix and match data sets to be statistically the same. *P*‐values less than 0.05 indicate that the mix and match data sets are statistically different from one another.

## RESULTS

**Figure 1:**
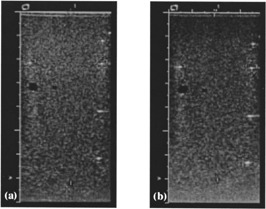
The match‐mix image pair producing the greatest absolute difference in the vertical distance accuracy measurement. (a) shows the match image for 8L5 transducer #3AB operated at 6 MHz, and (b) shows the mix image for this same transducer. The measured vertical distances for the mix and match conditions were 4.01 cm and 3.95 cm, respectively, producing a measurement difference of 0.06 cm.

**Figure 2:**
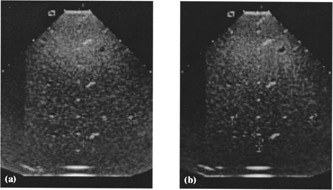
The match‐mix image pair producing the greatest absolute difference in horizontal distance accuracy measurement. (a) shows the match image for 4V1 transducer #3BV operated at H3 MHz, and (b) shows the mix image for this same transducer. (*H* denotes harmonic mode.) The measured horizontal distances for the mix and match conditions were 8.99 cm and 9.10 cm, respectively, producing a measurement difference of –0.11 cm.

**Figure 3:**
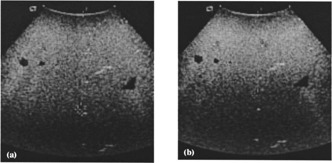
The match‐mix image pairs producing the greatest absolute difference in depth of penetration. (a) shows the match image for 8C4w transducer #3BA operated at H8 MHz, and (b) shows the mix image for this same transducer. The measured depths of penetration for the mix and match conditions were 6.02 cm and 5.2 cm, respectively, producing a measurement difference of 0.82 cm.

**Figure 4:**
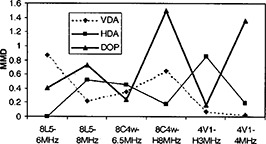
Graph of the mix‐match difference (MMD) absolute values for the vertical distance accuracy (VDA), horizontal distance accuracy (HDA), and depth of penetration (DOP) measurements made with all transducers and frequencies.

**Figure 5:**
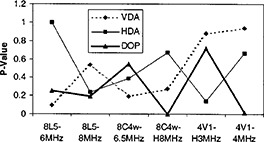
Graph of the *t*‐test *P*‐values for the vertical distance accuracy (VDA), horizontal distance accuracy (HDA), and depth of penetration (DOP) measurements made with all transducers and frequencies.

Numerical results for the VDA, HDA, and DOP are shown in Tables I–III, respectively. These tables show the measurement mean, standard deviation (SD), and error for each group of data corresponding to a specific transducer, frequency, and match or mix condition. For each pair of data groups, the MMD and *t*‐test *P*‐value are also shown. Each of these statistics is derived from ten match or mix measurements of each type. Figures 1–3 show pairs of images for the VDA, HDA, and DOP measurements, respectively. For each measurement type, the mix and match images obtained with the particular transducer that produced the largest measurement difference between the mix and match conditions is shown. Figures 4 and 5 show graphs of MMD and *t*‐test *P*‐values, respectively, for each phantom measurement as a function of transducer and frequency.

**Table I:** Vertical distance accuracy results (SD=standard deviation, MMD=mix‐match difference).

	Transducer and Frequency					
	8L5 6 MHz	8L5 8 MHz	8C4w 6.5 MHz	8C4w H8 MHz	4V1 H3MHz	4V1 4 MHz
Match Data						
mean (cm)	4.00	2.03	7.97	3.99	11.94	11.97
SD (cm)	0.01	0.01	0.02	0.01	0.04	0.03
error (%)	−0.08	1.30	−0.33	−0.28	−0.53	−0.25
Mix Data						
mean (cm)	3.99	2.02	7.97	4.00	11.94	11.97
SD (cm)	0.02	0.01	0.02	0.01	0.05	0.04
error (%)	−0.35	1.15	−0.41	−0.05	−0.50	−0.26
Match vs Mix						
MMD	0.87	0.22	0.35	−0.65	−0.07	0.03
*t*‐test *P*‐value	0.10	0.54	0.19	0.28	0.88	0.94

**Table II:** Horizontal distance accuracy results (SD=standard deviation, MMD=mix‐match difference).

	Transducer and Frequency					
	8L5 6 MHz	8L5 8 MHz	8C4w 6.5 MHz	8C4w H8 MHz	4V1 H3 MHz	4V1 4 MHz
Match Data						
mean (cm)	3.00	2.98	3.02	3.01	9.00	9.03
SD (cm)	0.02	0.02	0.02	0.02	0.05	0.06
error (%)	−0.01	−0.67	0.8	0.4	−0.01	0.29
Mix Data						
mean (cm)	3.00	2.99	3.01	3.02	9.04	9.04
SD (cm)	0.02	0.02	0.03	0.03	0.04	0.04
error (%)	−0.1	−0.33	0.43	0.57	0.41	0.4
Match vs Mix						
MMD	0.00	−0.52	0.45	−0.18	−0.85	−0.19
*t*‐test *P*‐value	1.00	0.24	0.39	0.67	0.14	0.66

**Table III:** Depth of penetration results (SD=standard deviation, MMD=mix‐match difference).

	Transducer and Frequency					
	8L5 6 MHz	8L5 8 MHz	8C4w 6.5 MHz	8C4w H8 MHz	4V1 H3MHz	4V1 4 MHz
Match Data						
mean (cm)	7.38	5.11	9.19	5.60	15.26	18.11
SD (cm)	0.13	0.13	0.22	0.31	0.24	0.04
Mix Data						
mean (cm)	7.33	5.01	9.14	5.21	15.30	18.17
SD (cm)	0.12	0.15	0.18	0.22	0.23	0.05
Match vs Mix						
MMD	0.41	0.73	0.24	1.50	−0.17	−1.36
*t*‐test *P*‐value	0.25	0.19	0.54	0.00	0.72	0.02

## DISCUSSION

### Vertical Distance Accuracy

The vertical distance accuracy results are shown in Table I. It can be seen that all measurement SD values range between 0.01 and 0.05 cm. Larger SD values indicate greater measurement variability and are undesirable. The largest SD value was recorded for the mix condition using the 4V1 transducer at H3 MHz. Comparing all sets of mix and match SDs obtained with the same transducer and frequency, it is seen that the mix SD is larger than the match SD in three of six cases, the match SD is never larger than the mix SD, and the two SD values are the same in three of six cases. A similar comparison of the error values shows that these range between −0.53% and +1.30%. Positive error values indicate measurements that are greater than the known true distance, and negative error values indicate measurements that are smaller than the known true distance. Obviously, error values that are closer to zero are desirable. The largest absolute error was recorded for the match condition using the 8L5 transducer at 8 MHz. Comparing all sets of mix and match errors obtained with the same transducer and frequency, it is seen that the mix error is larger that the match error in three of six cases, and the match error is larger than the mix error in three of six cases. The MMDs between the mix and match conditions for all transducers and frequencies are all less than 0.87. This is well below the threshold value of 2, suggesting that the mix and match data are not different. Also, all of the *t*‐test *P*‐values for all six transducer‐frequency pairs of mix and match data are greater than 0.10. This is above the threshold of 0.05, again suggesting that the mix and match data are not different. Considering all of these measures, no appreciable differences between the match and mix data are evident. This conclusion is also supported by the similarity of the images shown in Fig. 1. No appreciable differences between the images are evident.

### Horizontal Distance Accuracy

The horizontal distance accuracy measurement results are shown in Table II. It can be seen that all measurement SD values range between 0.02 and 0.06 cm. The largest SD value was recorded for the match condition using the 4V1 transducer at 4 MHz. Comparing all sets of mix and match SDs obtained with the same transducer and frequency, it is seen that the mix SD is larger than the match SD in two of six cases, the match SD is larger than the mix SD in two of six cases, and the two SD values are the same in two of six cases. A similar comparison of the error values shows that these range between −0.67% and +0.80%. The largest error value was recorded for the match condition using the 8C4w transducer at 6.5 MHz. Comparing all sets of mix and match errors obtained with the same transducer and frequency, it is seen that the mix error is larger than the match error in three of six cases, the match error is larger than the mix error in two of six cases, and the two SD values are the same in one of six cases. The MMDs between the mix and match conditions for all transducers and frequencies are all less than 0.85 in absolute value. This is well below the threshold value of 2, suggesting that the mix and match data are not different. Also, all of the *t*‐test *P*‐values for all six transducer‐frequency pairs of mix and match data are greater than 0.14. This is above the threshold of 0.05, again suggesting that the mix and match data are not different. Considering all of these measures, no appreciable differences between the match and mix data are evident. This conclusion is also supported by the similarity of the images shown in Fig. 2. No appreciable differences between the images are evident.

### Depth of Penetration

The depth of penetration (DOP) results are shown in Table III. It can be seen that all measurement SD values range between 0.04 and 0.31 cm. The largest SD value was recorded for the match condition using the 8C4w transducer at H8 MHz. Comparing all sets of mix and match SDs obtained with the same transducer and frequency, it is seen that the mix SD is larger than the match SD in two of six cases, and the match SD is larger than the mix SD in four of six cases. Since the DOP measurement is subjective, and no “correct” absolute depth of penetration values were available, no absolute error measurements could be made. However, the mean values can be compared, assuming that larger depths of penetration are “better.” Comparing all sets of mix and match mean values obtained with the same transducer and frequency, it is seen that the mix mean is larger that the match mean in two of six cases, and the match mean is larger than the mix mean in four of six cases. Considering all of the depth of penetration standard deviation and mean data discussed above, as seen previously for the vertical and horizontal distance accuracy measurements, no obvious differences between the matched and the mixed data sets are evident.

The MMDs between the mix and match conditions for all transducers and frequencies are all less than 1.50. This is below the threshold value of 2, suggesting that the mix and match data are not different. However, in two of the six cases the *t*‐test *P*‐values do indicate that differences between the mix and match data are present. These two cases involve the 4V1 transducer operating at 4 MHz (P‐value=0.02) and the 8C4w transducer operating at H8 MHz (P‐value=0.0008). In the case of the 4V1 transducer operating at 4 MHz, the *P*‐value is actually a symptom of a flaw in the study design. An operating frequency of 4 MHz allows the transducer to image to the greatest depth of the phantom (18 cm). Because DOP measurements were obtained by placing the scanner calipers at the top of the image and then at the maximum depth of penetration, small lateral errors in caliper placement causing them to lie in slightly different pixel columns can cause small errors, on the order of 1%, to creep into the data. This is reflected in the 4V1 at 4 MHz DOP data in Table III, where the mean values are about 1% greater than the maximum depth of 18 cm (lateral caliper placement errors will cause the measurement to be slightly larger than it should be). The standard deviation values of 0.04 cm and 0.05 cm are also artificially minimized compared to the other SD values reported in Table III, and are less than 0.5% of the maximum depth of 18 cm. These artificially reduced SD values are the root cause of the large MMD and *P*‐values. A different frequency or imaging mode should have been used instead of 4 MHz. These 4V1 at 4 MHz DOP data offer no information on the similarity or difference of the match and mix cases.

In the second case where statistical differences between mix and match data are seen (8C4w at H8 MHz), the mean DOP for the match data (5.60 cm) is greater than for the mix data (5.21 cm). The SD values for these two data sets (0.31 and 0.22 cm for the mix and match conditions, respectively) are similar to the other data (excluding the 4V1 at 4 MHz measurement as previously discussed), although 0.31 cm is the largest SD value recorded. The mix and match data sets obtained using the 8C4w transducer at 6.5 MHz are statistically indistinguishable.

### Complete Set of All Measurement Data

Considering all of the match and the mix data for all three measurements, and all transducers and frequencies, we conclude that the mix and match data are very comparable. There is no appreciable, systematic difference between the match and the mix data sets. This is the same conclusion we reached in our initial preliminary experiment.^5^ The two DOP cases where differences were suggested represent only two of the six DOP measurement comparisons, and only two of 18 total comparisons made for all three measurements. In one of these cases (4V1 at 4 MHz), the detected difference was artifactual, and was due to a study design flaw. No differences are suggested for the remaining 16 comparisons. In our experience, the more subjective DOP measurement is prone to larger variations than the vertical and horizontal distance accuracy measurements, and this is reflected in the SD data for the three tests. This conclusion regarding the practical equivalence of the mix and match data seems especially well supported in the context of ultrasound quality assurance. In this application, if we assume that the performance of matched scanner‐transducer pairs is better than for mixed pairs, the risk is that we are fooled into thinking that a transducer has a problem because of a biased mix QA measurement, when the equipment is actually operating normally. If a QA measurement indicates that a transducer falls out of the acceptable range of performance, our standard practice dictates that the equipment be re‐tested. If the re‐test is done using the matched scanner, any risk incurred by the possibility of appreciable differences between the mix and match situations is eliminated. An additional measurement made using the original scanner with its matched transducer could examine the possibility that the problem actually lies with that scanner, and not the transducer. Our recommended approach is to obtain match data whenever feasible, but to allow mix data to be obtained if QA practice efficiencies can be attained.

It is interesting also to note that of the 180 measurements obtained in each data set, five match measurements fell outside of our control limits, as did five mix measurements. Four of the “failed” measurements in each mix and match group were HDA or VDA measurements, all of which were within 0.1 mm of the control limit. One failed measurement in each group was a DOP measurement. The mix DOP and match DOP measurements exceeded their acceptance limits by 1.7% and 6.7%, respectively. Taken as a whole, the match and mix results are again quite comparable.

When making the measurements in this study, information in the images did not indicate whether they were produced with matched or mixed scanner‐transducer pairs, although no explicit effort to randomize the images was made. We felt that any bias introduced would be small compared to the bias produced by the practical QA situation in which the experienced operator learns the expected measurement values for each transducer and frequency combination. For example, we did previously report a study^7^ in which we measured the standard deviation of DOP measurements made in a completely blinded situation. The DOP standard deviation found in that study for single readers averaged about 0.3 cm, which is larger than most of the measurements presented in the current work. This does suggest that DOP standard deviation values in the current study may be somewhat smaller than they would be in a completely blinded situation. This is not felt to be a detriment in this study, since smaller standard deviations might tend to make true differences between the matched and mixed cases easier to detect. Also, as mentioned earlier, this is the practical situation when making these measurements for QA purposes. Examination of the data in Tables I–III reveals no obvious biases between the matched and mixed cases, and our overall conclusion that the matched and mixed measurements are very comparable also supports this.

The mix and match study reflects the ultrasound equipment's clinical use and demands. Not all of the ultrasound scanners at our institution have the same number of transducers or the same transducer types. A sonographer may be performing a particular examination that requires the use of a transducer not matched with the particular scanner they are using. The result is clinical image acquisition using a mixed scanner‐transducer combination. The study has provided some assurance of extremely similar performance of matched and mixed combinations. Even if match‐mix differences could be seen reliably in the phantom test images (and in our experience no systematic differences are evident), observable effects in clinical images are judged to be extremely unlikely.

In practice, allowing QA measurements to be obtained using mixed scanner‐transducer combinations allows our measurements to be obtained in a much more time efficient manner. Our largest US imaging location alone has ten scanners with 66 transducers, and keeping these transducers properly matched with the scanners when used by a rotating group of over 40 sonographers is nearly impossible. When initial mix measurements are allowed, QA testing can be done room by room, testing those US transducers available within that room. The time spent waiting to search for matched transducers in those US scanning rooms occupied by patients can then be avoided. Our endovaginal transducers in each imaging area are stored together with their appropriate disinfectant solution and prep supplies. Batch QA testing of like transducer types all on one scanner can reduce footwork, scanner time setup, and help keep imaging presets and techniques consistent for those transducer measurements. Any differences in like transducer performance can readily be appreciated as well. The overall approach to QA testing and scheduling becomes much more flexible and can be customized to suit departmental needs, with no degradation in utility of the QA program. Care must be taken, however, to assure that each US scanner is used to obtain a full set of QA measurements at least once during each QA cycle.

We expect that the results of this study are influenced by the particular phantom tests we included, and the specific procedure for each test. We included all three quantitative imaging tests that are routinely performed in our current US QA program. It is conceivable that differences scanner‐transducer combinations. It is therefore also recommended that this type of study be repearted as US equipment from new vendors is acquired.ets might be seen for certain QA tests and not for others. Figures 4 and 5 graph the MMD and *t*‐test *P*‐value for the three tests as functions of transducer and frequency. Inspection of these graphs indicates that none of the three tests shows greater or lesser equivalence between the mix and match data. It is recommended that this type of study be repeated whenever additional QA tests are added (e.g., axial and lateral spatial resolution), or when significant changes are made to our procedures (e.g., introducing automated analysis via computer). Similarly, it is conceivable that ultrasound equipment from different vendors will be more or less sensitive to mixed US scanner‐transducer combinations. It is therefore also recommended that this type of study be repeated as US equipment from new vendors is acquired.

## CONCLUSIONS

We observed no appreciable, systematic differences between overall mix and match measurements of vertical distance accuracy, horizontal distance accuracy, and depth of penetration. Although our statistical testing did indicate significant differences in 2 of the 18 data sets compared, we judge that the overall comparison of all of the data show that the mix and match measurements are very comparable. This is especially true in the context of US scanner QA. We recommend that match data be obtained where it is practical to do so for US QA purposes, but if this is not feasible, measurements from mixed scanner‐transducer combinations may be obtained, especially if QA program efficiencies may be obtained.
